# A chromosome-level genome assembly of the yellow-throated marten (*Martes flavigula*)

**DOI:** 10.1038/s41597-023-02120-3

**Published:** 2023-04-17

**Authors:** Xuesong Mei, Guangshuai Liu, Jiakuo Yan, Chao Zhao, Xibao Wang, Shengyang Zhou, Qinguo Wei, Shihu Zhao, Zhao Liu, Weilai Sha, Honghai Zhang

**Affiliations:** School of Life Sciences, Qufu Normal University, Qufu, 273165 Shandong China

**Keywords:** Genome, Sequencing

## Abstract

The yellow-throated marten (*Martes flavigula*) is a medium-sized carnivore that is widely distributed across much of Asia and occupies an extensive variety of habitats. We reported a high-quality genome assembly of this organism that was generated using Oxford Nanopore and Hi-C technologies. The final genome sequences contained 215 contigs with a total size of 2,449.15 Mb and a contig N50 length of 68.60 Mb. Using Hi-C analysis, 2,419.20 Mb (98.78%) of the assembled sequences were anchored onto 21 linkage groups. Merqury evaluation suggested that the genome was 94.95% complete with a QV value of 43.75. Additionally, the genome was found to comprise approximately 39.74% repeat sequences, of which long interspersed elements (LINE) that accounted for 26.13% of the entire genome, were the most abundant. Of the 20,464 protein-coding genes, prediction and functional annotation was successfully performed for 20,322 (99.31%) genes. The high-quality, chromosome-level genome of the marten reported in this study will serve as a reference for future studies on genetic diversity, evolution, and conservation biology.

## Background & Summary

The yellow-throated marten belongs to the genus *Martes* of the family Mustelidae and is named after its conspicuous yellow pelage on its chest and throat^1^. It is a voracious predator that feeds on several types of vertebrates, invertebrates, fruit, nectar, and food residue^2^. Unlike many mustelids, the marten generally moves in groups of two to three individuals^2^, which enables increased access to resources and reduces the risk of predation^3^. Given its preference for forested areas, it rarely appears in non-wooded environments^4^, as a consequence of which it may serve as a good indicator of forest ecosystem health. The marten performs several key roles in maintaining ecological balance, including spreading seeds^5^, and controlling the herbivore population size^6^, as a top-level predator in certain ecosystems^4^. The risk of extinction faced by the marten is low and the International Union for the Conservation of Nature (IUCN) classifies it as “Least Concern”^7^. However, rampant hunting, habitat loss, and other human activities pose substantial danger to the gradually decreasing marten populations^7^. Fortunately, certain protective measures, including legislation to counter these trends have been implemented in several countries, such as Myanmar^8^, Thailand^9^, South Korea^6^, and China^10^.

At present, research on the marten primarily focuses on its physical characteristics, behaviour, geographic range, and habitat. However, progress in molecular characterization, albeit slowly, has resulted in complete elucidation of its mitochondrial genome^11,12^, established phylogenetic relationships between species on the basis of mitochondrial and/or partial nuclear gene sequences^13–15^, and enabled population genetics analyses based on microsatellite markers^16,17^. Genetic and evolutionary studies on the marten have been limited by the sparse nature of available genomic resources. For instance, the marten is the only extant species of the genus *Martes* that is adapted for survival in areas spanning from boreal to equatorial regions and from sea-level to an altitude of 4,510 m^7^. The likelihood is that there is some genetic variation among populations of the marten occupying different habitats. Therefore, a meaningful analysis of population structure, and the molecular mechanisms of adaptive evolution among different marten populations at the genomic level will be highly valuable. We applied Oxford Nanopore and Hi-C technologies to generate a chromosome-level genome assembly of the marten, which will serve as a useful resource in evolutionary and population genetics studies on this animal, as well as in chromosome evolution studies on Carnivora.

## Methods

### Sampling and sequencing

The yellow-throated marten sample used for DNA and RNA sequencing was obtained from Chengdu, China. Muscle tissue was stored at −80 °C and used to construct Illumina, Nanopore, and Hi-C libraries. High molecular weight genomic DNA was extracted from muscle tissues using a Blood & Cell Culture DNA Midi Kit.

Short-insert-size (~400 bp) paired-end sequencing libraries were constructed using the Truseq Nano DNA HT Sample Preparation Kit and sequenced on the Illumina HiSeq X Ten platform to generate 150 bp paired-end reads. These yielded 1.58 billion reads, 236.83 Gb of raw sequence data, which covered 96.70-fold of the genome assembly (Table 1, Table S1). Nanopore libraries were constructed and sequenced on the PromethION sequencer. In total, 27.76 million reads, 264.40 Gb of raw sequence data were obtained, which was 107.96-fold coverage of the genome assembly (Table 1, Table S2). The mean read length and the N50 length were 9.53 kb and 17.43 kb, and the longest read covered 204.65 kb (Table S2). Hi-C libraries were constructed using *MboI* restriction enzyme and sequenced on the Illumina NovaSeq6000 platform in 150 bp PE mode. As a result, 257.31 Gb of Hi-C reads were obtained, which covered 105.07-fold of the genome assembly (Table 1, Table S3).

**Table 1:** Statistics of sequencing data generated in this study.

Types	Method	Raw data (Gb)	Average read length (bp)	Coverage (X)
Genome	Illumina	236.83	150	96.70
Genome	Nanopore	264.40	9,525	107.96
Genome	Hi-C	257.31	150	105.07
RNA	Illumina	60.43	150	—

Additionally, RNA was extracted from seven tissues of the marten, including testis, stomach, kidney, pancreas, heart, spleen, and intestine. Transcriptome sequencing was performed on the Illumina Novaseq6000 platform, which yielded a total of 60.43 Gb of raw reads (Table 1, Table S4).

### Genome size and heterozygosity estimation

**Fig. 1:**
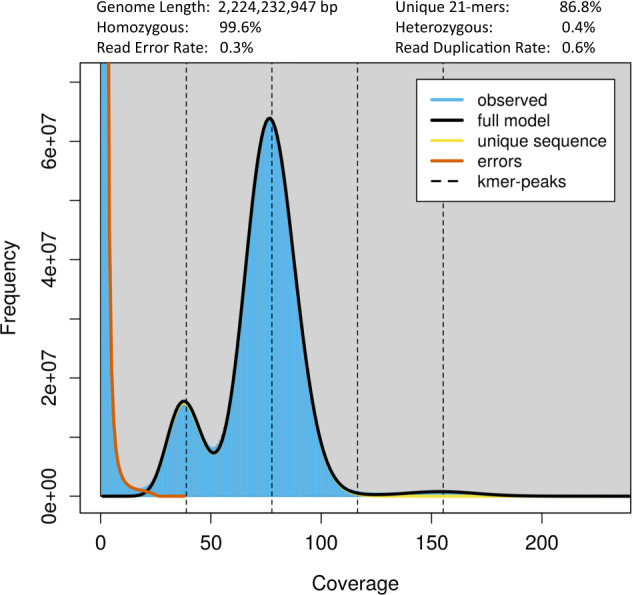
The 21-mer frequency distribution analysis for the marten genome based on Illumina paired-end reads. The observed 21-mer frequency distribution is shown in blue, whereas the fitted model is shown as a black line. The unique and putative error k-mer distributions are plotted in yellow and red, respectively.

Raw genomic Illumina sequencing reads were filtered using Fastp v0.12.6^18^ to remove adaptors, duplications, and low-quality reads. The clean reads were subsequently used to estimate genome size, heterozygosity, and repeat content based on 21-mer frequency distribution analysis using Jellyfish v2.3.0^19^ and GenomeScope v2.0^20^. This resulted in the identification of 205,236,235,649 21-mers with a depth of 77 (Table S5). We therefore estimated that the genome of the marten is approximately 2,224.23 Mb in size, with a heterozygosity of 0.40% and a repeat content of 13.16% (Fig. 1, Table S5).

### De novo assembly of the marten genome

Sequencing data generated from the Nanopore platform were corrected (parameters: “reads_cutoff: 1k,seed_cutoff: 19k”) and assembled (parameters: default) using NextDenovo v2.0-beta.1 (https://github.com/Nextomics/NextDenovo). Further improvement in the accuracy of the assembly was ensured by performing four rounds of self-correction and three rounds of consensus correction using ONT reads and Illumina short reads with Nextpolish v1.0.5^21^. The finally assembled genome was 2449.15 Mb in size with 215 contigs and a contig N50 of 68.60 Mb (Table 2). These findings closely mirror the genome size of *Martes zibellina* (2,420.68 Mb), a closely related species of the marten^22^. Further genome assembly summary statistics were computed using Gfastats v1.3.3^23^ (Table 2).

**Table 2:** Summary statistics of the genome assembly.

Genome Assembly		Genome Assembly (Hi-C version)	
Key	Value	Key	Value
Contig number	215	Scaffold number	130
Contig N50 (bp)	68,603,938	Scaffold N50 (bp)	143,113,826
Contig auN	75,438,269.27	Scaffold auN	143,573,002.40
Contig L50	12	Scaffold L50	7
Average contig length (bp)	11,391,392.89	Average scaffold length (bp)	18,839,676.70
Largest contig length (bp)	165,781,393	Largest scaffold length (bp)	219,655,006
Total contig length (bp)	2,449,149,471	Total scaffold length (bp)	2,449,157,971
GC content (%)	41.73	GC content (%)	41.73
Gaps	0	Gaps	8,500

### Chromosomal-level scaffolding

**Fig. 2:**
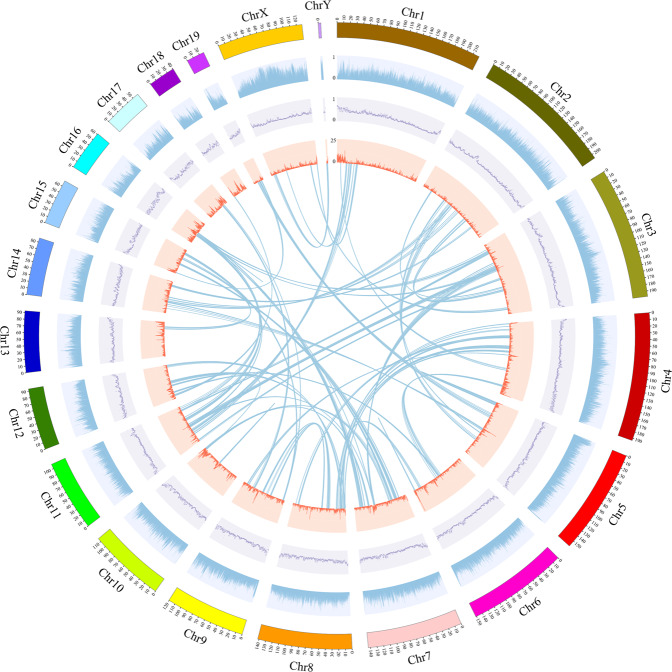
Features of the marten genome. The tracks from outside to inside are 21 chromosomes, repeat sequences abundance (blue), GC content (purple), gene abundance (red), collinear regions (each line connects a pair of homologous genes). The figure used for circos plot was generated using TBtools^59^.

**Fig. 3:**
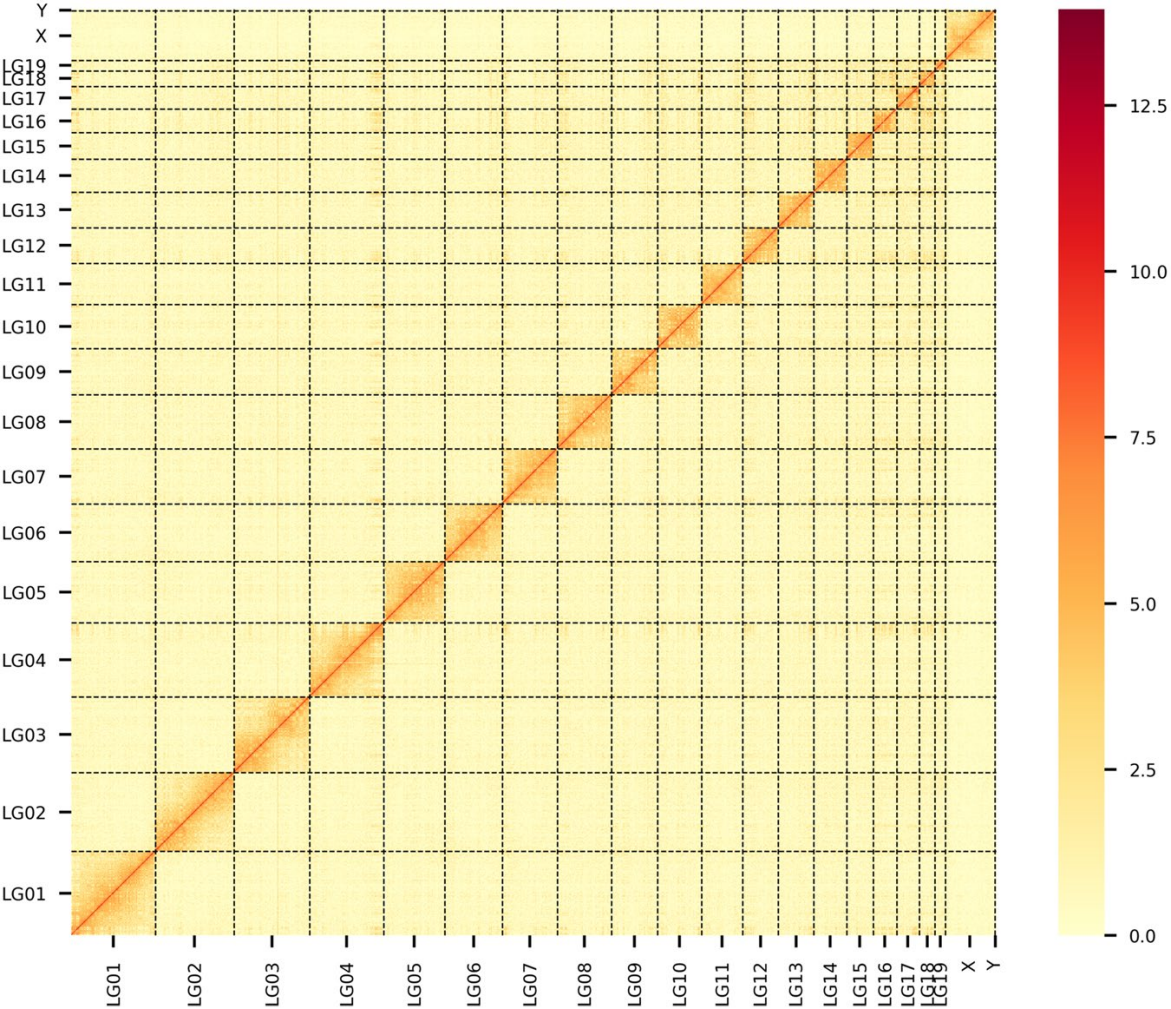
Genome-wide all-by-all Hi-C interaction among 21 chromosomes of the marten. The heatmap indicates that intra-chromosome interactions (blocks on the diagonal line) are stronger than inter-chromosome interactions. The shading gradient on the right represents the intensity of chromosomal interactions, which ranges from white (low) to red (high).

Chromosome-level scaffolding was performed by Hi-C analysis at the Genome Center of Grandomics (Wuhan, China). The raw Hi-C data were primarily filtered using Hi-C-Pro v2.8.0^24^. Subsequently, post quality control with Fastp, the clean Hi-C data were mapped to the genome assembly of the marten using Bowtie2 v2.3.2^25^ to get the unique mapped paired-end reads. As a result, 608.63 million uniquely mapped pair-end reads were obtained (Table S6), of which 83.19% were valid interaction pairs (Table S7). Combined with the valid Hi-C data, LACHESIS^24^ was applied to produce a chromosomal-level genome. We further adjusted the misassembled contigs manually based on the interaction strength among the contigs and a linkage map using Juicebox^26^. The final outcome entailed 2,419.20 Mb (98.78%) of assembled sequences that were anchored and orientated onto 21 chromosomes, ranging from 3.97 Mb to 219.65 Mb in length (Fig. 2, Table 3). Subsequently, the software ggplot2 in the R package was used to generate a genome-wide Hi-C heatmap to evaluate the quality of the chromosomal-level genome. The heatmap of chromosome crosstalk illustrated that the chromosomal-level genome was complete and robust (Fig. 3).

**Table 3:** Statistics of the chromosomal-level genome.

Chr ID	Chr length (bp)	Contig num	GC content (%)
LG01	219,654,706	4	41.08
LG02	206,652,498	3	39.00
LG03	198,243,934	2	39.53
LG04	193,413,641	4	41.75
LG05	159,516,868	14	40.34
LG06	151,133,825	3	40.22
LG07	143,113,326	6	41.46
LG08	141,978,359	5	43.06
LG09	120,416,158	2	41.58
LG10	115,536,597	4	42.80
LG11	107,115,054	6	40.74
LG12	93,305,887	3	43.13
LG13	92,516,399	6	42.83
LG14	86,383,869	3	43.31
LG15	68,875,827	7	42.98
LG16	61,452,323	9	47.18
LG17	59,306,037	2	46.47
LG18	40,719,113	3	48.48
LG19	27,362,197	1	44.34
X	128,529,105	14	40.48
Y	3,973,310	5	41.12
Total	2,419,199,033	106	41.65

### Genome quality assessment

Complementary methods were employed to evaluate the quality of genome assembly. First, the Illumina reads and Nanopore reads were aligned to the marten genome using BWA v0.7.12-r1039^27^ and Minimap2 v2.17^28^, respectively. The results showed that 99.85% of the Illumina reads and 99.74% of the Nanopore reads could be mapped to the genome, with a coverage rate of 99.87% and almost 100%, respectively (Table S8, Table S9). Second, the completeness of the genome was evaluated by BUSCO v4.0.5 with a core gene set, referring to mammalia_odb10^29^. As a result, 93.37% (8,614 of 9,226) of the complete BUSCO genes were identified, of which, 93.03% (8,583 of 9,226) were single copy and 0.34% (31 of 9,226) were duplicated (Fig. S1). Third, Merqury v1.3^30^ was used to assess the consensus quality value (QV) and k-mer completeness of the genome assembly, which were found to be 43.75 and 94.95%, respectively (Table S10, Fig. S2).

### Repeat annotation

Homology-based and *ab initio* prediction methods were used to identify repetitive sequences in the marten genome. The homology-based analysis was performed using RepeatMasker v4.1.0^31^ with the Repbase database^32^. For *ab initio* prediction, RepeatModeler v2.0.1^33^ was utilized to construct a *de novo* repeat library, which was subsequently employed to predict repeats with RepeatMasker. We identified 973.18 Mb of repetitive sequences, accounting for 39.74% of the marten genome (Table 4). Among these, long interspersed elements (LINE) that accounted for 26.13% of the whole genome were the most abundant (Table 4). These results are supported by similar findings in published mustelids genomes^22,34,35^.

**Table 4:** Statistics of repetitive elements in the marten genome.

TE subtype	Length (bp)	% in genome
DNA	67,465,275	2.76
LINE	639,768,860	26.13
SINE	72,767,201	2.97
LTR	123,377,969	5.04
Other	11,324,428	0.45
Unknown	58,472,885	2.39
Total	973,176,618	39.74

### Prediction and functional annotation of protein-coding genes

We predicted protein-coding genes in the marten genome through integrating three different strategies: *ab initio* prediction, homology-based prediction, and transcriptome-based prediction. First, Augustus v2.5.5^36^, GlimmerHMM v3.0.4^37^, Geneid v1.4.4^38^, and Genscan v1.0^39^ were adopted to *ab initio* gene prediction with internal gene models. Second, protein sequences of seven species including, *Bos taurus*, *Canis lupus familiaris*, *Enhydra lutris*, *Homo sapiens*, *Mustela erminea*, *Mustela putorius furo*, and *Mus musculus*, as the templates of protein homology-based prediction were downloaded and aligned against the marten genome using TblastN v2.2.26^40^ with an E-value ≤ 1e−5. The potential gene structure of each alignment was then predicted by GeneWise v2.4.1^41^. Third, transcriptome data were aligned to the marten genome with TopHat v2.1.1^42^ and the gene structures were predicted by Cufflinks v2.2.1^43^. Finally, a non-redundant gene set was generated via integration of the three respective annotation files that were assigned different weights (*ab initio* prediction was “1”, homology-based prediction was “5”, and transcriptome-based prediction was “10”) in EVidenceModeler v1.1.1. PASA v2.3.3 was used to update the gene models by identifying untranslated regions to generate a final annotation^44^.

Functional annotation of the protein-coding genes was accomplished using eggNOG-Mapper v2^45^, a tool that enables rapid functional annotations of novel sequences on the basis of pre-computed orthology assignments, against the EggNOG v5.0 database^46^.

**Fig. 4:**
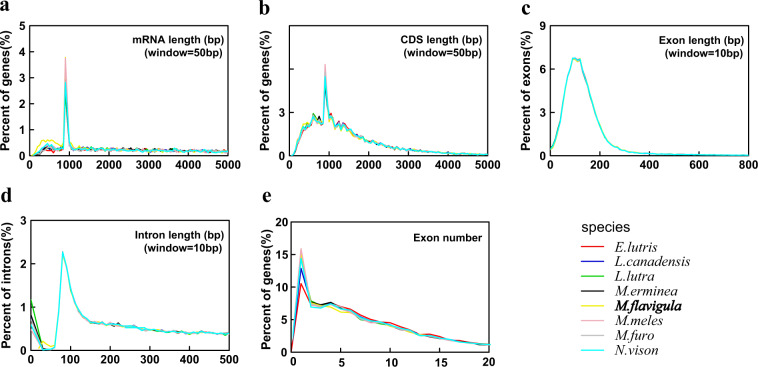
The comparisons of gene elements in the marten genome with seven other mustelids. (**a**) mRNA length distribution and comparison with seven other mustelids. (**b**) CDS length distribution and comparison with seven other mustelids. (**c**) Exon length distribution and comparison with seven other mustelids. (**d**) Intron length distribution and comparison with seven other mustelids. (**e**) Exon number distribution and comparison with seven other mustelids.

Overall, we obtained 20,464 protein-coding genes in the marten genome, of which, 20,322 (99.31%) were successfully annotated. Additionally, we compared the distribution of mRNA length, coding DNA sequence (CDS) length, exon length, intron length and exon number in the marten genome with that of seven other mustelids, including, *Enhydra lutris*, *Lontra canadensis*, *Lutra lutra*, *Mustela erminea*, *Meles meles*, *Mustela putorius furo*, and *Neovison vison* (Table 5, Fig. 4). The results revealed a higher percentage of shorter mRNA in the marten genome than that in the genomes of the seven other mustelids (Fig. 4a). Further, short intronic lengths (about 0~75 bp) in the marten genome had a distribution pattern that was distinct from the seven other mustelids (Fig. 4d). One of the possible reasons is that there is slight deviation in the results of genome assembly and/or annotation between different species.

**Table 5:** The comparisons of gene elements in the marten genome with seven other mustelids.

species	Number	Average mRNA length (bp)	Average CDS length (bp)	Average exon length (bp)	Average intron length (bp)	Average exons per gene
*E.lutris*	19,327	48,788.57	1,734.41	173.84	4,808.60	9.98
*L.canadensis*	20,305	47,440.83	1,694.47	175.67	4,765.80	9.65
*L.lutra*	20,764	49,572.42	1,694.13	178.33	4,924.05	9.50
*M.erminea*	20,921	49,263.24	1,699.35	179.58	4,918.25	9.46
*M.flavigula*	20,464	44,272.10	1,715.32	179.22	4,459.60	9.57
*M.meles*	21,063	48,654.45	1,691.12	180.60	4,935.63	9.36
*M.furo*	20,794	48,195.52	1,703.36	179.11	4,799.60	9.51
*N.vison*	20,409	48,353.15	1,704.29	178.19	4,825.90	9.56

## Data Records

The genomic Illumina sequencing data were deposited in the Sequence Read Archive at NCBI SRR21452075^47^. The genomic Nanopore sequencing data were deposited in the Sequence Read Archive at NCBI SRR21426791^48^. The transcriptome Illumina sequencing data were deposited in the Sequence Read Archive at NCBI SRR21460068-SRR21460074^49–55^. The Hi-C sequencing data were deposited in the Sequence Read Archive at NCBI SRR21430408^56^. The final chromosome assembly were deposited in the GenBank at NCBI JAODOS000000000^57^. The final chromosome assembly, gene structure annotation, repeat annotation, and gene functional prediction were deposited in the Figshare database^58^.

## Technical Validation

### DNA quantification and qualification

DNA degradation and contamination was monitored on 1% agarose gels. DNA purity was detected using NanoDrop One UV-Vis spectrophotometer. DNA concentration was measured by Qubit Fluorometer.

### RNA quantification and qualification

RNA degradation and contamination was monitored on 1% agarose gels. RNA concentration was measured by Qubit Flurometer. RNA integrity was assessed using Agilent 2100 Bioanalyzer.

**Quality filtering of Illumina data**

To make sure the reads reliable in the following analyses, we used Fastp to elevate the quality of raw reads generated from the Illumina platform. The data were filtered out as follows:

1. removing the reads with more than 10% of Ns;
2. removing the reads with a quality score less than 20 for 20% of bases;
3. removing the reads with adapter sequences;
4. removing the reads with duplications.

## Supplementary information

Supplementary materials

## Data Availability

No specific code or script was used in this work. The commands used in the processing were all executed according to the manuals and protocols of the corresponding bioinformatics software.
