# Supplementary material for: A chromosome-level genome assembly of the yellow-throated marten (Martes flavigula)
Source: Sci Data. 2023 Apr 17;10:216. doi: 10.1038/s41597-023-02120-3 (PMC10110515; doi:10.1038/s41597-023-02120-3)
Supplement: Supplementary file 1 — Supplementary materials [file 41597_2023_2120_MOESM1_ESM.pdf]

Supplementary contents	Page number
Table S1. Statistics of genomic Illumina sequencing data.	1
Table S2. Statistics of Nanopore sequencing data.	1
Table S3. Statistics of Hi-C sequencing data.	1
Table S4. Statistics of transcriptome sequencing data.	1
Table S5. Statistics of genome size estimation by 21-mer analysis.	1
Table S6. Statistics of alignment results of clean paired-end reads.	2
Table S7. Statistics of valid paired-end reads.	2
Table S8. Statistics of alignment results of Illumina short-reads.	2
Table S9. Statistics of alignment results of Nanopore long-reads.	3
Table S10. Statistics of genome quality assessment results by Merqury.	3
Fig S1. The results of genome quality assessment by BUSCO method.	3
Fig S2. K-mer spectra copy number plot.	3

Table S1. Statistics of genomic Illumina sequencing data.

Total reads	Total bases (bp)	Clean reads	Clean bases (bp)	Q20 (%)	Q30 (%)	GC (%)
1,578,886,764	236,833,014,600	1,551,923,696	216,829,874,157	96.88	91.63	42.74

Table S2. Statistics of Nanopore sequencing data.

Library	Total pass reads bases / bp	Total pass reads number	Pass reads mean length / bp	Pass reads max length / bp	Pass reads N50 length /bp
L1	28,045,956,211	3,149,647	8,904	204,654	17,534
L2	45,152,203,426	4,054,854	11,135	194,957	21,332
L3	31,814,921,972	3,612,794	8,806	191,090	16,591
L4	65,007,591,254	7,325,336	8,874	196,511	16,043
L5	64,699,586,550	5,816,543	11,123	203,038	19,346
L6	29,678,172,580	3,798,332	7,813	201,868	12,621
Total	264,398,431,993	27,757,506	9,525	204,654	17,428

Table S3. Statistics of Hi-C sequencing data.

Raw paired-end reads	Clean paired-end reads	Clean bases (bp)	Clean paired-end reads rate (%)	Clean Q30 bases rate (%)
1,701,675,478	1,679,998,582	251,847,035,907	98.73	92.73

Table S4. Statistics of transcriptome sequencing data.

Sample name	Raw reads	Ran bases (bp)	Read length (bp)	Q30 (%)
Testis	23,214,499	6,964,349,700	150; 150	95.19; 92.08
Intestine	26,307,794	7,892,338,200	150; 150	95.35; 92.79
Stomach	28,976,575	8,692,972,500	150; 150	95.72; 92.73
Kidney	32,946,932	9,884,079,600	150; 150	95.52; 91.93
Pancreas	25,518,799	7,655,639,700	150; 150	95.99; 93.85
Heart	26,299,305	7,889,791,500	150; 150	95.06; 92.29
Spleen	38,181,303	11,454,390,900	150; 150	95.17; 92.17

Table S5. Statistics of genome size estimation by 21-mer analysis.

K-mer	K-mer number	K-mer depth	Genome size (Mb)	Heterozygosity rate	repeat content
21	205,236,235,649	77	2,224.23	0.40%	13.16%

Table S6. Statistics of alignment results of clean paired-end reads.

Type	Value
Clean paired-end reads	839,999,291
Unmapped paired-end reads	18,928,365
Unmapped paired-end reads rate (%)	2.25
Paired-end reads with singleton	93,521,934
Paired-end reads with singleton rate (%)	11.13
Multi mapped paired-end reads	118,920,457
Multi mapped ratio (%)	14.16
Unique mapped paired-end reads	608,628,535
Unique mapped ratio (%)	72.46

Table S7. Statistics of valid paired-end reads.

Type	Value
Unique mapped paired-end reads	608,628,535
Dangling end paired-end reads	5,106,904
Self-circle paired-end reads	272,472
Dumped paired-end reads	92,930,426
Valid paired-end reads	506,310,404
Valid rate (%)	83.19
Valid reads of unique mapping (%)	60.28

Table S8. Statistics of alignment results of Illumina short-reads.

Type	Value
Map reads	1,556,127,709
Map rate (%)	99.85
Paired reads	1,551,923,696
Paired map reads	1,548,780,764
Properly paired reads	1,504,275,802
Properly map rate (%)	96.93
Average sequencing depth (X)	87.01
Coverage (%)	99.87
Coverage at least 5X (%)	99.83
Coverage at least 10X (%)	99.79
Coverage at least 20X (%)	99.68

Table S9. Statistics of alignment results of Nanopore long-reads.

Type	Value
Map reads	29,956,156
Map rate (%)	99.74
Average sequencing depth (X)	103.3
Coverage (%)	100
Coverage at least 5X (%)	99.97
Coverage at least 10X (%)	99.94
Coverage at least 20X (%)	99.87

Table S10. Statistics of genome quality assessment results by Merqury.

k-mer completeness (%)	QV	Error rate
94.95	43.75	4.21885e-05

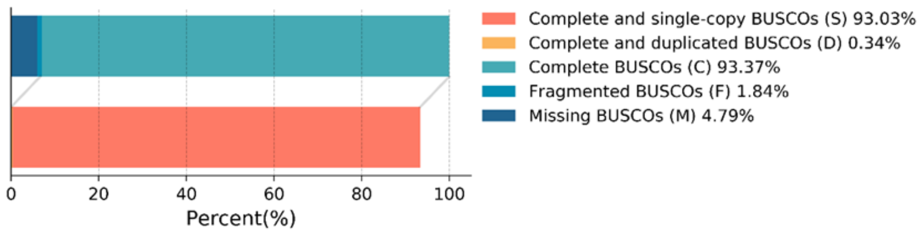

Fig S1. The results of genome quality assessment by BUSCO method.

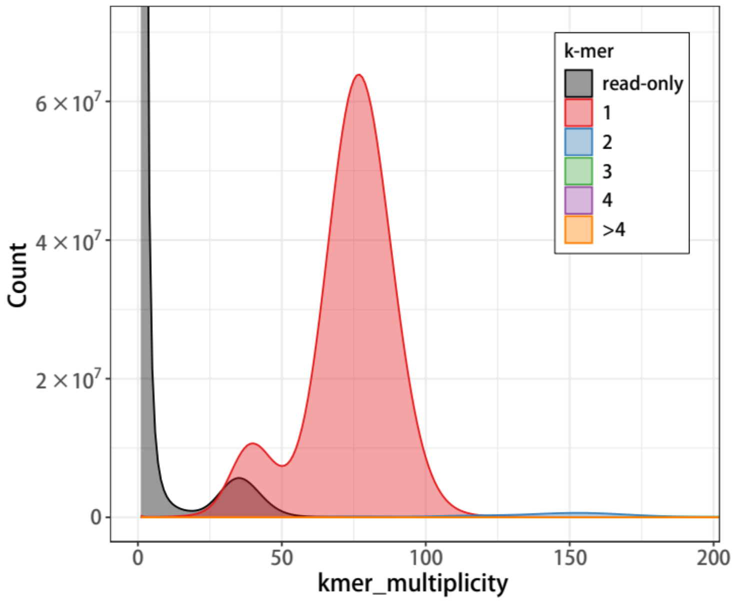

Fig S2. K-mer spectra copy number plot. Colors in the plot represent the number of times each k-mer is found in the genome assembly. Spectral distribution is computed based on the Illumina paired-end reads.
